# Vaborbactam: Spectrum of Beta-Lactamase Inhibition and Impact of Resistance Mechanisms on Activity in Enterobacteriaceae

**DOI:** 10.1128/AAC.01443-17

**Published:** 2017-10-24

**Authors:** Olga Lomovskaya, Dongxu Sun, Debora Rubio-Aparicio, Kirk Nelson, Ruslan Tsivkovski, David C. Griffith, Michael N. Dudley

**Affiliations:** The Medicines Company, San Diego, California, USA

**Keywords:** vaborbactam, meropenem-vaborbactam, KPC, major porins OmpK35 and OmpK36

## Abstract

Vaborbactam (formerly RPX7009) is a new beta-lactamase inhibitor based on a cyclic boronic acid pharmacophore. The spectrum of beta-lactamase inhibition by vaborbactam and the impact of bacterial efflux and permeability on its activity were determined using a panel of strains with beta-lactamases cloned from various classes and a panel of Klebsiella pneumoniae carbapenemase 3 (KPC-3)-producing isogenic strains with various combinations of efflux and porin mutations. Vaborbactam is a potent inhibitor of class A carbapenemases, such as KPC, as well as an inhibitor of other class A (CTX-M, SHV, TEM) and class C (P99, MIR, FOX) beta-lactamases. Vaborbactam does not inhibit class D or class B carbapenemases. When combined with meropenem, vaborbactam had the highest potency compared to the potencies of vaborbactam in combination with other antibiotics against strains producing the KPC beta-lactamase. Consistent with broad-spectrum beta-lactamase inhibition, vaborbactam reduced the meropenem MICs for engineered isogenic strains of K. pneumoniae with increased meropenem MICs due to a combination of extended-spectrum beta-lactamase production, class C beta-lactamase production, and reduced permeability due to porin mutations. Vaborbactam crosses the outer membrane of K. pneumoniae using both OmpK35 and OmpK36, but OmpK36 is the preferred porin. Efflux by the multidrug resistance efflux pump AcrAB-TolC had a minimal impact on vaborbactam activity. Investigation of the vaborbactam concentration necessary for restoration of meropenem potency showed that vaborbactam at 8 μg/ml results in meropenem MICs of ≤2 μg/ml in the most resistant engineered strains containing multiple mutations. Vaborbactam is a highly active beta-lactamase inhibitor that restores the activity of meropenem and other beta-lactam antibiotics in beta-lactamase-producing bacteria, particularly KPC-producing carbapenem-resistant Enterobacteriaceae.

## INTRODUCTION

Beta-lactam antibiotics are among the most useful classes of antibiotics to treat bacterial infections due to their spectra of activity, mode of action, and excellent safety profile. Among them, carbapenems have a particular clinical utility due to their resistance to hydrolysis by numerous beta-lactamases; however, the recent dissemination of carbapenemases that inactivate nearly all beta-lactams (including carbapenem antibiotics) threatens the loss of this class. Carbapenemases include serine beta-lactamases from Ambler classes A and D (1) and all metallo-beta-lactamases from class B (2, 3). In many locations around the world, including the United States, class A Klebsiella pneumoniae carbapenemases (KPC) represent the most prevalent carbapenemases (4–6). Infections caused by KPC-producing bacteria have been associated with increased health care costs and increased lengths of stay, as well as frequent treatment failures, with mortality rates varying from 22% to 72% (7–9). The *in vitro* characterization of KPC-producing strains has established that the bacteria evolved a multifactorial path to resistance, often combining the production of a carbapenemase with mutations that alter the expression or function of porins or efflux proteins (10–14).

The use of beta-lactam–beta-lactamase inhibitor (BLI) combinations is an effective strategy to overcome beta-lactamase-mediated resistance to beta-lactam antibiotics. However, older BLIs, such as clavulanic acid, tazobactam, and sulbactam (15), do not inhibit class A carbapenemases. Avibactam, the first member of the diazabicyclo-octane class, has a broader spectrum of beta-lactamase inhibition, including inhibition of many class A, class C, and some class D enzymes, than older BLIs (16, 17). Although avibactam was originally developed to address the dissemination of class A extended-spectrum beta-lactamases (ESBLs) and class C enzymes and was specifically optimized for activity against these beta-lactamases (18), it was subsequently found to inhibit the KPC carbapenemase (19). The potent activity of ceftazidime-avibactam against KPC-producing Enterobacteriaceae has been well documented in numerous studies (20, 21), and data from uncontrolled clinical series have shown its clinical efficacy (22) but also failures and relapses with ceftazidime-avibactam treatment (23), including the development of resistance during or following therapy (24, 25). This highlights the importance of the continued development of novel beta-lactam–beta-lactamase inhibitor combinations.

**FIG 1:**
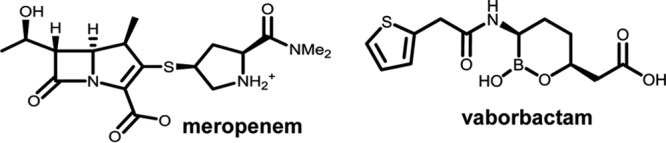
Chemical structures of meropenem and vaborbactam. Me_2_, dimethyl.

Vaborbactam (formerly RPX7009; Fig. 1), a new BLI based on a cyclic boronic acid pharmacophore, was discovered during a program specifically focused on targeting KPC carbapenemases. The biochemical, microbiological, and pharmacological properties of candidate BLIs were optimized for use in combination with a carbapenem antibiotic. Vaborbactam emerged from this effort and is being developed in combination with meropenem for the treatment of serious Gram-negative bacterial infections, including those due to KPC-producing carbapenem-resistant Enterobacteriaceae (CRE) (26).

This communication provides a detailed characterization of the spectrum of beta-lactamase inhibition by vaborbactam and the impact of efflux and uptake on restoration of the potency of meropenem. A panel of engineered strains of Escherichia coli and K. pneumoniae producing various beta-lactamases and an isogenic set of KPC-producing strains of K. pneumoniae with various combinations of efflux and porin mutations were used in these studies.

## RESULTS

### Vaborbactam is a broad-spectrum inhibitor of diverse class A and class C beta-lactamases with potent inhibitory activity against KPC and other class A carbapenemases.

The profile of beta-lactamase inhibition by vaborbactam was evaluated using a panel of engineered strains of E. coli expressing diverse beta-lactamase genes coding for four molecular classes of beta-lactamases. Meropenem MICs against the strains producing various carbapenemases (class A carbapenemases KPC-2/KPC-3, SME, and NMC-A; class D carbapenemase OXA-48; class B carbapenemases VIM and NDM) ranged from ≤0.125 μg/ml to 16 μg/ml, whereas the meropenem MIC against the vector-only control strain, ECM6704, was ≤0.03 μg/ml (Table 1). As expected, the strains producing ESBLs (SHV, TEM, CTX-M) or class C enzymes (CMY, chromosomal AmpC from Enterobacter cloacae) had sensitivity to meropenem similar to that of parent strain ECM6704. Vaborbactam at 4 μg/ml decreased the meropenem MICs against the strains producing class A carbapenemases to the level for the vector-only control strain. Meropenem MICs against the strains producing OXA-48 or class B carbapenemases were not decreased.

**TABLE 1:** MICs of ceftazidime and aztreonam alone or in combination with BLIs against the panel of engineered E. coli strains producing various cloned beta-lactamases^*a*^

Strain	Beta-lactamase	Class	Antibiotic MIC (μg/ml) in the absence or presence of BLIs									
			CAZ	CAZ + VAB	CAZ + TZB	CAZ + CLA	ATM	ATM + VAB	ATM + TZB	ATM + CLA	MEM	MEM + VAB
ECM6704	None		≤0.125	≤0.125	≤0.125	≤0.125	≤0.125	≤0.125	≤0.125	≤0.125	≤0.03	≤0.03
ECM6701	KPC-2	A-CARB	4	≤0.125	4	2	32	≤0.125	16	16	2	≤0.03
ECM6702	KPC-3	A-CARB	16	≤0.125	16	8	32	≤0.125	16	16	2	≤0.03
ECM6706	SME-2	A-CARB	1	≤0.125	≤0.125	0.25	>128	0.25	4	16	16	≤0.03
ECM6696	NMC-A	A-CARB	0.5	≤0.125	0.25	0.25	64	≤0.125	8	8	1	≤0.03
ECM6718	SHV-5	A-ESBL	8	0.5	≤0.125	≤0.125	16	1	≤0.125	≤0.125	≤0.03	≤0.03
ECM6698	SHV-12	A-ESBL	32	2	≤0.125	≤0.125	32	4	≤0.125	≤0.125	≤0.03	≤0.03
ECM6699	SHV-18	A-ESBL	8	0.5	≤0.125	≤0.125	16	1	≤0.125	≤0.125	≤0.03	≤0.03
ECM6713	TEM-10	A-ESBL	128	16	0.25	0.25	16	4	≤0.125	≤0.125	≤0.03	≤0.03
ECM6714	TEM-26	A-ESBL	128	2	≤0.125	0.25	8	2	≤0.125	≤0.125	≤0.03	≤0.03
ECM6695	CTX-M-3	A-ESBL	1	≤0.125	≤0.125	≤0.125	4	≤0.125	≤0.125	≤0.125	≤0.03	≤0.03
ECM6693	CTX-M-14	A-ESBL	1	≤0.125	≤0.125	≤0.125	4	≤0.125	≤0.125	≤0.125	≤0.03	≤0.03
ECM6694	CTX-M-15	A-ESBL	4	≤0.125	≤0.125	≤0.125	8	0.25	≤0.125	≤0.125	≤0.03	≤0.03
ECM6692	DHA-1	C	8	0.25	≤0.125	8	2	0.25	≤0.125	2	≤0.03	≤0.03
ECM6691	MIR-1	C	32	0.5	8	32	32	1	16	32	≤0.03	≤0.03
ECM6705	FOX-5	C	32	8	32	32	2	0.5	2	2	≤0.03	≤0.03
ECM6715	AmpC-ECL (P99-like)	C	16	0.25	1	16	16	0.5	2	16	≤0.03	≤0.03
ECM6700	CMY-2	C	16	0.25	0.5	16	8	0.25	1	8	≤0.03	≤0.03
ECM6697	OXA-2	D	1	1	0.25	≤0.125	≤0.125	ND	ND	ND	≤0.03	≤0.03
ECM6712	OXA-10	D	≤0.125	≤0.125	≤0.125	≤0.125	≤0.125	ND	ND	ND	≤0.03	≤0.03
ECM6716	OXA-48	D-CARB	≤0.125	≤0.125	≤0.125	≤0.125	≤0.125	ND	ND	ND	0.125	0.125
ECM6703	NDM-1	B	>128	>128	>128	>128	≤0.125	≤0.125	≤0.125	≤0.125	16	16
ECM6711	VIM-1	B	128	128	128	128	≤0.125	≤0.125	≤0.125	≤0.125	1	1

a All beta-lactamase inhibitors were tested at a fixed concentration of 4 μg/ml. BLIs, beta-lactamase inhibitors; CAZ, ceftazidime; ATM, aztreonam; MEM, meropenem; VAB, vaborbactam; TZB, tazobactam; CLA, clavulanic acid; ND, not done; A-CARB, class A carbapenemase; D-CARB, class D carbapenemase.

In order to fully characterize the spectrum of inhibition by vaborbactam, the activities of ESBL-labile agents ceftazidime and aztreonam were tested against a panel of beta-lactamase-producing strains alone and in combination with 4 μg/ml of vaborbactam, tazobactam, or clavulanic acid. In the absence of vaborbactam, the ceftazidime and aztreonam MICs against strains producing class A carbapenemases ranged from 0.5 to 16 μg/ml and from 32 to >128 μg/ml, respectively. As was seen with meropenem, vaborbactam strongly enhanced the activity of both antibiotics against these strains. In the presence of vaborbactam at 4 μg/ml, the MICs for both antibiotics were ≤0.125 μg/ml, the same as that seen for the strain containing the vector alone, indicating complete inhibition of beta-lactamase activity (Table 1).

Vaborbactam also restored the activity of ceftazidime and aztreonam against various ESBL-producing strains. It had potency comparable to that of tazobactam and clavulanic acid against CTX-M (CTX-M-3, CTX-M-14, CTX-M-15)-producing strains, but it was generally less potent against SHV (SHV-5, SHV-12, SHV-18) and TEM (TEM-10, TEM-26) producers (Table 1). In strains producing class C cephalosporinases, ceftazidime and aztreonam MICs decreased from 8 to 32 μg/ml and from 2 to 32 μg/ml, respectively, for the antibiotics alone to 0.25 to 8 μg/ml and to 0.25 to 1 μg/ml, respectively, for the antibiotics with vaborbactam at 4 μg/ml. Checkerboard experiments using aztreonam confirmed the higher potency (8- to 32-fold) of vaborbactam at reducing the MICs of antibiotics against strains producing KPC (and other class A carbapenemases) than against strains producing ESBLs (in particular, SHV/TEM) and class C beta-lactamases (see Table S3 in the supplemental material). Among the tested class D enzymes (OXA-2, OXA-10, OXA-48), only OXA-2 production resulted in a measurable increase in the ceftazidime MIC (from ≤0.125 to 1 μg/ml). The MIC for ECM6607 expressing OXA-2 remained 1 μg/ml in the presence of vaborbactam 4 μg/ml, indicating that OXA-2, similar to OXA-48, is not inhibited by this BLI. Vaborbactam did not have any inhibitory activity against the metallo-beta-lactamases NDM-1 and VIM-1 (Table 1). Evaluation of strains expressing additional metallo-beta-lactamases (multiple IMPs, SPM, CcrA, L1) confirmed this result (Table S4).

These studies demonstrate that vaborbactam is a broad-spectrum inhibitor of class A and class C beta-lactamases with particularly potent inhibitory activity against class A carbapenemases, such as KPC.

### Vaborbactam restores the potency of meropenem against engineered strains of K. pneumoniae with increased meropenem MICs due to the combination of ESBL or class C beta-lactamase production and reduced permeability due to porin mutations.

While hydrolysis by carbapenemases is the major mechanism of carbapenem resistance in clinical isolates, low-level resistance to carbapenems in Enterobacteriaceae can occur when some class A or class C noncarbapenemases are produced by strains with mutations resulting in reduced outer membrane permeability (27, 28). The activity of meropenem alone and in combination with vaborbactam against a panel of engineered strains of K. pneumoniae with or without permeability defects that produced selected class A or class C beta-lactamases was evaluated. KPC- and OXA-48-producing strains were used as positive and negative controls, respectively, for restoration of the potency of meropenem (Table 2). Only plasmids that carried KPC-2 and KPC-3 were able to confer a modest meropenem MIC increase (from ≤0.06 to 0.5 μg/ml) compared to the MIC for wild-type strain K. pneumoniae KPM1001 (ATCC 43816) (Table S2). When the same plasmids with these beta-lactamase genes were transformed into isogenic strain KPM1176 (Table S2), which overexpressed the *acrAB-tolC* operon, had *ompK35* downregulated due to a mutation in the *ramR* gene, and contained a loss-of-function mutation in *ompK36*, the meropenem MICs increased compared to those for the corresponding KPM1001 transformants; KPC- and OXA-48-producing strains each had MICs of 64 μg/ml, which corresponds to a 128-fold MIC increase compared to that for the vector-only control strain. As expected, vaborbactam enhanced the activity of meropenem against KPC-producing strains but not OXA-48-producing strains. An increase in the vaborbactam concentration was associated with a further reduction of the meropenem MIC for KPC-producing derivatives of KPM1176 (data not shown). CTX-M-15, CMY-2, and AmpC-ECL (P99-like) production but not SHV-12 production in KPM1176 was associated with a 4- to 8-fold increase in the meropenem MIC, but vaborbactam at 4 μg/ml reduced the MICs back to the level for the vector-only control strain (Table 2).

**TABLE 2:** MICs of meropenem alone or in combination with vaborbactam against isogenic engineered strains of K. pneumoniae strains producing various beta-lactamases^*a*^

Cloned beta-lactamase	Host strain KPM1001			Host strain KPM1176		
	Strain producing beta-lactamase	MIC (μg/ml)		Strain producing beta-lactamase	MIC (μg/ml)	
		MEM	MEM + VAB		MEM	MEM + VAB
pUCP24	KPM1116	≤0.06	≤0.06	KPM1950	0.5	0.25
KPC-2	KPM1113	0.5	≤0.06	KPM1931	64	2
KPC-3	KPM1049	0.5	≤0.06	KPM2840	64	2
CTX-M-15	KPM1114	≤0.06	≤0.06	KPM1943	2	0.5
SHV-12	KPM1115	≤0.06	≤0.06	KPM1945	0.5	0.25
CMY-2	KPM1045	≤0.06	≤0.06	KPM1948	2	0.5
AmpC-ECL	KPM1956	≤0.06	≤0.06	KPM1959	4	1
OXA-48	KPM1939	≤0.06	≤0.06	KPM1941	64	64

a Vaborbactam was used at a fixed concentration of 4 μg/ml. MEM, meropenem; VAB, vaborbactam. Based on sequence analysis (GenBank accession no. CP009208), KPM1001 (ATCC 43816) is a wild-type strain of K. pneumoniae containing full copies of *acrAB*, *ompK35*, and *ompK36* and no mutations in known regulators of these genes; hence, their level of expression is the same as that in the wild type. KPM1176 is an isogenic mutant (Table S2) that contains a mutation in the gene *ramR* (a frameshift from amino acid 46). This mutation is responsible for overexpression of the *acrAB* operon and for downregulation of the gene *ompK35* encoding the corresponding porin (see Table 5 and Table S5 in the supplemental material). KPM1176 also carries the loss-of-function mutation in the gene *ompK36* (a frameshift from amino acid 266).

### The reduction in beta-lactam MICs for KPC-producing strains by vaborbactam is greatest when vaborbactam is paired with a carbapenem rather than other classes of beta-lactams.

The effect of the vaborbactam concentration on the reduction in the MICs for several carbapenems (meropenem, biapenem, ertapenem, tebipenem, and imipenem) as well as ceftazidime, aztreonam, and cefepime against an engineered KPC-3-producing strain of K. pneumoniae KPM1271 (which has the KPM1001 and KPM1026a background and which is characterized by wild-type expression of the major efflux pump AcrAB-TolC and the outer membrane porins OmpK35 and OmpK36 [see Table 5]) was determined. The potency of vaborbactam was expressed as the minimum potentiation concentration (MPC) required to reduce the carbapenem MIC by 16-fold (MPC_16_) in a KPC-producing strain (KPM1271). In general, vaborbactam MPC_16_ values were lower when vaborbactam was tested with carbapenems than when it was tested with other beta-lactams; the vaborbactam MPC_16_ ranged from 0.03 to 0.06 μg/ml for all the carbapenems, with the exception of imipenem (MPC_16_, 0.25 μg/ml). For cefepime, aztreonam, and ceftazidime, the vaborbactam MPC_16_ values were 0.125 to 0.25 μg/ml (Table 3). These results indicate that vaborbactam has the most potent activity against this KPC-producing strain when it is tested in combination with most carbapenems compared with its activity when it is tested in combination with other beta-lactam antibiotics.

**TABLE 3:** Effect of vaborbactam concentration on MICs of antibiotics against the engineered KPC-3-producing strain of K. pneumoniae, KPM1271^*a*^

Antibiotic	MIC (μg/ml) in the presence of the following concn of vaborbactam (μg/ml):								MPC_16_ (μg/ml)
	0	0.015	0.03	0.06	0.125	0.25	0.5	1	
Meropenem	16	2	1	0.5	0.25	0.25	0.125	≤0.06	0.03
Biapenem	16	4	4	1	1	0.5	0.25	0.25	0.06
Ertapenem	32	8	2	1	0.5	0.25	0.25	≤0.06	0.03
Tebipenem	32	8	2	1	0.5	0.25	0.125	0.125	0.03
Imipenem	8	8	4	2	1	0.5	0.5	0.5	0.25
Aztreonam	32	32	32	32	2	1	0.25	0.125	0.125
Ceftazidime	64	64	64	32	8	4	1	1	0.25
Cefepime	4	4	2	0.5	0.125	0.06	0.03	0.03	0.125

a MPC, minimal potentiation concentration; MPC_16_, the MPC of vaborbactam required to reduce the MIC by 16-fold. KPM1271 was constructed by conjugating plasmid pKpQIL, which carries the gene for KPC-3, from clinical isolate KP1074 into KPM1026a, the streptomycin-resistant mutant of wild-type strain KPM1001 (ATCC 43816 with wild-type expression of a major efflux pump AcrAB-TolC and functional porins OmpK35 and OmpK36).

### The potency of vaborbactam is similar against strains producing KPC-2 or KPC-3.

The effect of the vaborbactam concentration on the meropenem MIC was determined in KPM1176 derivatives KPM1931 and KPM2840, producing KPC-2 and KPC-3, respectively (Table 4). This particular host strain with permeability defects (see above) was used to increase the sensitivity of the potentiation assay. The meropenem MICs and the concentration-response of vaborbactam inhibitory potency were comparable in these two strains, indicating that meropenem and vaborbactam have similar activities against strains producing KPC-2 or KPC-3.

**TABLE 4:** Effect of vaborbactam concentration on meropenem MICs for engineered strains of K. pneumoniae producing cloned KPC-2 and KPC-3 beta-lactamases^*a*^

Strain	Beta-lactamase	Meropenem MIC (μg/ml) in the presence of the following concn of vaborbactam (μg/ml):							
		0	1	2	4	8	16	32	64
KPM1941	KPC-2	128	64	16	4	2	1	0.5	0.5
KPM2840	KPC-3	128	64	8	4	2	1	0.5	0.5

a Plasmids containing cloned *bla*_KPC-2_ and *bla*_KPC-3_ were transformed into strain KPM1176, in which *acrAB* is upregulated and *ompK35* is downregulated and which carries a loss-of-function mutation in *ompK36* (see Table 5 and Table S5 in the supplemental material).

### OmpK35 and OmpK36 outer membrane porins, but not efflux, are important determinants of vaborbactam uptake in K. pneumoniae.

To investigate the effect of increased efflux and various porin mutations on the microbiological potency of vaborbactam, a set of isogenic strains of K. pneumoniae with various combinations of efflux and porin mutations was constructed. The natural plasmid pKpQIL, which carries KPC-3, was introduced into each strain by conjugation using the clinical strain KP1074 (now ATCC BAA-2814) as a donor.

### (i) Parent carbapenemase-negative strains and meropenem.

The MICs of meropenem and minocycline against the parent strains that lack KPC are shown in Table 5. Minocycline, a known substrate of AcrAB-TolC, was included as a control for AcrAB activity. Inactivation of *ompK35* or *ompK36* individually did not affect the meropenem MICs; the meropenem MICs for KPM2600 (Δ*ompK35*) and KPM2592 (Δ*ompK36*), or KPM2040 (*ompK36*_2067, with a frameshift at amino acid 54) were unchanged from the MIC for KPM1026a (the wild-type parental strain; MIC = 0.03 μg/ml). Inactivation of both *ompK35* and *ompK36* (KPM2613) increased the meropenem MIC 4-fold (to 0.125 μg/ml). Overexpression of *acrAB* due to a mutation in the negative regulator *ramR* did not affect the MIC of meropenem (KPM1026a versus KPM1027; Table 5 and S5). Of note, the same mutation also led to the downregulation of *ompK35* (Table S5), indicating that overexpression of *acrAB* did not have an effect on the meropenem MIC even in combination with a decreased level of expression of *ompK35*. The complete inactivation of *ompK35* in the strain overexpressing *acrAB* did not affect the MIC of meropenem (KPM2610 versus KPM1027); however, the overexpression of *acrAB* in the strain with both *ompK35* and *ompK36* inactivated increased the meropenem MIC 4-fold to 0.5 μg/ml (KPM2966 versus KPM2613). Inactivation of *ompK36* in the strain with a reduced level of expression of *ompK35* and overexpression of *acrAB* (due to the mutation in the *ramR* gene) increased the MIC of meropenem 8-fold to 0.25 μg/ml (KPM2658 and KPM1176 versus KPM1027). These data indicate that the overexpression of *acrAB* caused a 2- to 4-fold increase in the MIC of meropenem in the absence of both major porins and that the meropenem MIC could be increased up to 16-fold when double porin mutations were combined with increased efflux.

**TABLE 5:** Effect of efflux and porins on activities of antibiotics against a panel of isogenic strains of K. pneumoniae with efflux and porin mutations

Strain^*a*^	Genotype	Effect of:			MIC^*b*^ (μg/ml)	
		OmpK35	OmpK36	AcrAB	MER	MIN
KPM1026a^*c*^	Wild type	Wild type (1)	Wild type (1)	Wild type (1)	0.03	1
KPM2600	Δ*ompK35*	Nonfunctional	Wild type	Wild type	0.03	2
KPM2592	Δ*ompK36*	Wild type	Nonfunctional	Wild type	0.03	1
KPM2040	*ompK36_2067*^*d*^	Wild type	Nonfunctional	Wild type	0.03	1
KPM2613	*ompK36_2067* Δ*ompK35*	Nonfunctional	Nonfunctional	Wild type	0.125	2
KPM2966	*ramR^*e*^ ompK36_2067* Δ*ompK35*	Nonfunctional	Nonfunctional	Upregulated	0.5	8
KPM1027^*f*^	*ramR*^*g*^	Downregulated (0.1)	Wild type (0.9)	Upregulated (3.1)	0.03	8
KPM2610	*ramR* Δ*ompK35*	Nonfunctional	Wild type	Upregulated	0.03	8
KPM2658	*ramR* Δ*ompK36*	Downregulated	Nonfunctional	Upregulated	0.25	8
KPM1176^*h*^	*ramR ompK36_1176*	Downregulated (0.1)	Nonfunctional	Upregulated (3.9)	0.25	8

a All strains contained chromosomal SHV enzyme, encoded by *bla*_SHV-24_.

b MER, meropenem; MIN, minocycline.

c KPM1026a is a streptomycin-resistant mutant of wild-type strain KPM1001 (ATCC 43816). It contains a functional *acrAB* operon and functional *ompK35* and *ompK36* genes. Normalized expression of *acrB*, *ompK35*, and *ompK36* in this strain was set equal to 1 (the normalized relative level of expression is shown in parentheses). The minocycline MIC for KPM1026a reflects the wild-type level of *acrAB* expression.

d Insertion of an A reside at nucleotide 160 of *ompK*36, causing a frameshift from amino acid 54 of OmpK36.

e A G490T substitution in *ramR* created TAA at amino acid 164.

f KPM1027 is a derivative of KPM1026a selected on tigecycline. It has a mutation in the negative regulator gene *ramR* and as a result has the *acrAB* operon overexpressed ∼3-fold and the *ompK35* gene downregulated ∼10-fold relative to their levels of expression in KPM1026a. Expression of *ompK36* in KPM1027 is unchanged relative to that in KPM1026a (the normalized relative level of expression is shown in parentheses).

g An 8-bp insertion in *ramR* causing a frameshift from amino acid 46.

h KPM1176 was selected from KPM1027 on meropenem at 0.25 μg/ml. It has a mutation in the *ompK36* gene that results in a frameshift in OmpK36 from amino acid 266. Similar to KPM1027, it has *acrAB* overexpressed and *ompK35* downregulated ∼3- to 4-fold and ∼10-fold, respectively, relative to their levels of expression in KPM1026a (the normalized relative level of expression is shown in parentheses). The functional status and expression levels of *acrAB*, *ompK35*, and *ompK36* in other strains were inferred from strain construction, *ramR* sequence analysis, and minocycline MICs.

### (ii) KPC-3-producing strains and meropenem.

The MICs of meropenem alone or with various concentrations of vaborbactam against isogenic KPC-producing strains are shown in Table 6. The meropenem MIC for the wild-type strain KPM1271 (in which both porins are produced and which has a normal level of *acrAB*), producing the KPC-3 beta-lactamase, was 16 μg/ml. Overexpression of *acrAB* or inactivation of *ompK35* did not have any measurable effect on the MIC of meropenem (KPM1271 versus KPM1272 and KPM2601). Inactivation of *ompK36* alone increased the meropenem MIC by 2-fold (KPM1271 versus KPM2599 and KPM2067); note that no such increase was seen in the absence of KPC (Table 5). Inactivation of *ompK35* in the strain that did not have a functional *ompK36* increased the meropenem MIC by 8-fold (from 32 to 256 μg/ml; KPM2067 versus KPM2631). Notably, overexpression of *acrAB* did not have any effect on the meropenem MIC even when both major porins were nonfunctional (KPM2631 versus KPM2818 or KPM2631 versus KPM2965). The kinetic parameters of meropenem efflux by AcrAB-TolC are unknown; however, if the pump is saturated at a concentration of meropenem that is lower than that required to inhibit the growth of the KPC-producing strain, the impact of efflux on the meropenem MIC is expected to be negligible.

**TABLE 6:** Effects of various concentrations of vaborbactam on meropenem MICs in isogenic KPC-3-producing strains of K. pneumoniae with efflux and porin mutations

KPC-3^*a*^-containing strain	Parent strain	Description	Meropenem MIC (μg/ml) in the presence of the following concn of vaborbactam (μg/ml):													MPC_max_^*b*^ (μg/ml)
			0	0.06	0.125	0.25	0.5	1	2	4	8	16	32	64	128	
Isogenic laboratory strains																
KPM1271	KPM1026a	Wild type	16	0.25	0.25	0.06	0.06	0.06	0.06	0.06	0.06	0.06	0.06	0.06	0.06	0.25
KPM2601	KPM2600	*ompK35* inactivated	16	2	1	0.5	0.125	0.06	0.06	0.06	0.06	0.06	0.06	0.06	0.06	1
KPM2599	KPM2592	*ompK36* inactivated	32	16	16	8	8	8	0.5	0.25	0.125	0.06	0.06	0.06	0.06	16
KPM2067	KPM2040	*ompK36* inactivated	32	32	16	16	16	4	1	0.5	0.125	0.06	0.06	0.06	0.06	16
KPM2631	KPM2613	*omp35* and *ompK36* inactivated	256	256	256	128	128	64	16	4	1	0.5	0.25	0.25	0.125	128
KPM2965	KPM2966	*acrAB* upregulated, *ompK35* and *ompK36* inactivated	256	256	256	128	128	64	32	8	2	1	0.5	0.5	0.25	128
KPM1272	KPM1027	*acrAB* upregulated, *ompK35* downregulated	16	8	2	2	0.5	0.25	0.06	0.06	0.06	0.06	0.06	0.06	0.06	2
KPM2818	KPM2658	*acrAB* upregulated, *ompK35* downregulated, *ompK36* inactivated	256	256	256	128	64	32	16	8	2	1	1	0.5	0.5	64
Clinical strains and derivatives																
KP1074	NA^*c*^	*ompK35* inactivated,^*d*^ OmpK36 is the same as in KP1004 but has the GD repeat^*e*^	128	128	128	64	64	64	8	1	0.5	0.25	0.125	0.125	0.125	32
KPM2644^*f*^	KP1074	KP1074 Δ*ompK36*, *ompK35* and *ompK36* inactivated	512	512	512	512	256	256	128	8	2	1	0.5	0.5	0.5	32
KP1004	NA	*ompK35* inactivated, full-length *ompK36*	32	4	4	2	0.5	0.125	0.03	0.03	0.03	0.03	0.03	0.03	0.03	2

a All strains produced KPC-3 and TEM-1, encoded by genes carried on plasmid pKpQIL. Both KPM1026a derivatives and clinical isolates also produced a chromosomal SHV enzyme, encoded by *bla*_SHV-24_ and *bla*_SHV-11_, respectively.

b MPC_max_, maximum potentiating concentration of the BLI required to reduce the meropenem MIC to the level seen in the parent strain that lacks KPC, corresponding to complete inhibition of KPC.

c NA, not available.

d A frameshift in the OmpK35 sequence at amino acid 42.

e The GD repeat is a duplication of two amino acids, Gly134 and Asp135, located within the L3 internal loop and associated with reduced susceptibility to carbapenems due to constriction of the channel (29).

f KPM2644 was constructed as follows. First, pKpQIL was cured from KP1074. Second, the resulting strain was used to select for an Sm^r^ mutant (on 200 μg/ml of streptomycin) to facilitate conjugation experiments. Third, *ompK36* was disrupted in KPM1308, giving rise to KPM2617. Finally, plasmid pKpQIL was conjugated from KP1074 into KPM2617.

### (iii) KPC-3-producing strains and meropenem-vaborbactam.

MPC_max_ corresponds to the concentration of vaborbactam that is required to achieve the maximal reduction of the meropenem MIC and was the parameter used to evaluate the potency of vaborbactam (maximal potentiation; Table 6). Inactivation of *ompK35* alone increased the vaborbactam MPC_max_ 4-fold (KPM2601 versus KPM1271), while inactivation of *ompK36* alone increased the vaborbactam MPC_max_ 64-fold. These results indicate that both porins are involved in the passage of vaborbactam across the outer membrane of K. pneumoniae, but OmpK36 plays the major role (KPM2599 and KPM2067 versus KPM1271). Inactivation of *ompK35* in the strain that already lacked *ompK36* (KPM2631 versus KPM2067) increased the MPC_max_ 4-fold, consistent with some contribution of OmpK35 to vaborbactam uptake but a contribution less significant than that of OmpK36.

Strains KPM1272 and KPM1271 were used to assess the effect of the *ramR* mutation. Inactivation of *ramR* (KPM1271) resulted in both *acrAB* overexpression and *ompK35* downregulation. The MPC_max_ was 8-fold higher for KPM1272 than for KPM1271; however, the MPC_max_ for KPM1272 was only 2-fold higher relative to that for KPM2601, the mutant that lacked OmpK35. In addition, no effect of AcrAB overexpression on vaborbactam potency was seen in the strains that lacked (or had low levels of expression of) both OmpK35 and OmpK36 (KPM2631 versus KPM2818 and KPM2631 versus KPM2965). These data indicate that while the function of either of the major porins affects the potency of vaborbactam, AcrAB-mediated efflux has a minimal effect.

### (iv) Effect of Gly134 and Asp135 duplication in OmpK36 on vaborbactam inhibition in KPC-producing strains of K. pneumoniae.

Strains of K. pneumoniae that carry a variant of OmpK36 with a duplication of two amino acids, Gly134 and Asp135 (GD repeat), are frequently reported in clinical settings (12, 13, 29, 30). We determined the MICs of meropenem alone or with various concentrations of vaborbactam against KP1074 (which expresses OmpK36 with the GD repeat) and its derivative, KPM2644, which carried a deletion of the *ompK36* gene. Another clinical isolate, KP1004, was also included in this experiment. Both strain KP1074 and strain KP1004 carry the same KPC plasmid and have nonfunctional OmpK35 due to the same mutation, a frameshift at amino acid 42, and nearly identical OmpK36 amino acid sequences other than the GD repeat in the OmpK36 of KP1074.

The meropenem MIC for KP1074 (128 μg/ml) was 4-fold higher than that for KP1004 (32 μg/ml) (Table 6). While these strains are not truly isogenic, the GD repeat in KP1074 is the most likely reason for this difference. The MPC_max_ was 16-fold higher for KP1074 than for KP1004, implicating that the GD repeat reduced the potency of vaborbactam in this strain as well. Deletion of the *ompK36* gene from KP1074 increased the MIC of meropenem 4-fold to 512 μg/ml (KPM2644 versus KP1074; Table 6); however, almost no concomitant decrease in vaborbactam potency was observed, with the MPC_max_ remaining unchanged. Thus, it appears that OmpK36 with a functionally constricted inner channel due to the 2-amino-acid duplication in the L3 loop still maintains some, albeit a decreased level of, meropenem uptake. Uptake is further reduced by deletion of *ompK36*, where vaborbactam appears to be unable to pass through the narrow channel of OmpK36 altered because of the GD duplication.

## DISCUSSION

An important feature of the drug discovery program that resulted in vaborbactam was the early choice of a partner antibiotic, which allowed a specific focus on the most relevant beta-lactamase-mediated resistance mechanism. From the start of the discovery effort, we intended to combine a carbapenem antibiotic with a novel BLI that was specifically optimized for the inhibition of KPC, the most prevalent carbapenemase in the United States and many other geographic regions (31). The main focus was on the simultaneous optimization of both the kinetics of KPC inhibition and microbiological activity, i.e., enhancing the potency of carbapenems against KPC-producing strains. Since carbapenems are stable to hydrolysis by the majority of class A and class C enzymes, we focused on optimizing the potency against a specific target rather than attempting to achieve potent broad-spectrum inhibition of multiple enzymes.

KPC-2 and KPC-3 represent the most prevalent KPC variants reported worldwide (32–35). Ceftazidime hydrolysis is ∼10-fold more efficient with KPC-3 than with KPC-2 (36, 37). A recent study by Shields and colleagues (38) found that median ceftazidime-avibactam MICs were higher against KPC-3 variants than against KPC-2 variants. In contrast, the studies described here indicated that there is no difference between the KPC-2 and KPC-3 hydrolysis of meropenem. Similarly, the similar concentration-effect response for the restoration of meropenem activity by vaborbactam against engineered strains producing either KPC-2 or KPC-3 observed in this study is also consistent with the biochemistry of meropenem hydrolysis.

In panels of engineered strains expressing cloned genes for various beta-lactamases, it was demonstrated that vaborbactam is an inhibitor of various class A and class C beta-lactamases and that its potency in the restoration of the activity of beta-lactams varied depending on the specific enzyme. Vaborbactam was the most potent against KPC-producing strains and the least potent against TEM- and SHV-producing strains. Since vaborbactam is being developed in combination with meropenem, which is stable to hydrolysis by the majority of beta-lactamases, including TEM and SHV, it is its high potency against KPC and other class A carbapenemases that constitutes its most relevant attribute as a BLI. Indeed, recent studies using large collections of clinical isolates producing the KPC carbapenemase demonstrated that vaborbactam significantly increased the activity of carbapenems against these strains (31, 39, 40). Recent biochemical experiments using nitrocefin as a substrate demonstrated that in the case of KPC-2, CTX-M-15, and P99 AmpC, vaborbactam behaved as a two-step tight-binding inhibitor that forms an initial noncovalent complex with an enzyme (characterized by affinity constant *K*) followed by enzyme inactivation upon covalent bond formation (characterized by rate constant *k*_2_). Moreover, vaborbactam inactivated various beta-lactamases with a comparable efficiency (measured as the *k*_2_/*K* ratio): the inactivation efficiency was ∼0.007 μM^−1^ s^−1^ for KPC-2 and ∼0.02 μM^−1^ s^−1^ for CTX-M-15 and P99 AmpC. However, the off rate of dissociation (*k*_off_) of the vaborbactam–beta-lactamase complex varied dramatically (50- to 200-fold) for different enzymes: *k*_off_ was 0.000017 s^−1^ (residence time [RT] = 992 min) for KPC-2, whereas *k*_off_ was 0.0009 s^−1^ (RT = 19 min) and 0.056 s^−1^ (RT = 3 min) for CTX-M-15 and P99 AmpC, respectively. In the case of the SHV-12 and TEM-43 enzymes, vaborbactam exhibited a fast-on–fast-off behavior with no signs of enzyme inactivation (41). It is conceivable that the low off rate of vaborbactam for KPC plays an important role in the high potency of this compound in enhancing the activities of antibiotics against KPC-producing strains determined in microbiological experiments. By the same token, the low potency with which vaborbactam enhances the activity of antibiotics against SHV- and TEM-producing strains might be driven by its inability to form a stable inhibitory complex with these enzymes.

Vaborbactam was more potent in restoring the activity of carbapenems (with the exception of imipenem) than that of other substrates of KPC. The results of microbiological experiments are in good agreement with those of biochemical experiments that demonstrated that the *K_i_* of KPC inhibition was ∼15-fold lower when carbapenems were used as KPC substrates than when the model cephalosporin substrate nitrocefin was used as a KPC substrate (R. Tsivkovski and O. Lomovskaya, unpublished observations). The KPC-mediated hydrolysis of imipenem was inhibited by vaborbactam with a potency ∼10-fold lower (the highest *K_i_*) than that of other carbapenems. On a biochemical level, the lower that the ability of a beta-lactamase to inactivate a given substrate is, the easier it is to inhibit such hydrolysis. Notably, meropenem appears to be a poorer substrate for KPC than nitrocefin and imipenem: the enzymatic efficiency of meropenem hydrolysis (*k*_cat_/*K_m_*) is ∼4-fold and ∼6-fold lower than that of the hydrolysis of nitrocefin and imipenem, respectively (36, 37). These data underscore the importance of optimization of a BLI with a specific partner beta-lactam.

While the inhibitory activity of vaborbactam against class A ESBLs and class C cephalosporinases is generally less important when it is paired with a stable carbapenem, the inhibition of these beta-lactamases may be important in some non-carbapenemase-producing, carbapenem-resistant strains of Enterobacteriaceae with porin mutations (27, 28). Data obtained from studies using engineered strains indicate that vaborbactam restores the activity of meropenem against these strains. The greater potency of vaborbactam for KPC enzymes compared to that for other enzymes becomes important, in that bacterial cells rarely produce KPC alone; most frequently, a single cell simultaneously produces other beta-lactamases, in addition to KPC (38, 42). However, since it is KPC that hydrolyzes meropenem, an inhibitor with a higher affinity for KPC will mainly target this enzyme and not other beta-lactamases that hydrolyze some beta-lactams but not meropenem.

Numerous studies have demonstrated the contribution of porin mutations to the phenotype of reduced susceptibility to carbapenems in clinical isolates of Enterobacteriaceae, irrespective of the presence of a carbapenemase (11, 28, 43, 44). In addition, the major efflux pump AcrAB has been implicated in reduced carbapenem susceptibility in clinical strains of Enterobacteriaceae (45). High-level resistance to carbapenems is associated with the production of a carbapenemase in the strains that lack major porins due to mutations or downregulation of the corresponding genes and have increased efflux (10, 11, 46). The impact of porin and efflux mutations on the potency of vaborbactam and meropenem-vaborbactam against isogenic mutants of KPC-producing K. pneumoniae demonstrated that, similar to carbapenems, vaborbactam can cross the outer membrane using both the OmpK35 and OmpK36 porins; however, unlike carbapenems that can use both porins with a similar efficiency (inactivation of OmpK35 or OmpK36 individually does not affect the carbapenem MIC), OmpK36, which has a smaller channel than OmpK35, appears to play a more significant role in the passage of vaborbactam across the outer membrane than OmpK35. This conclusion was based on the finding that inactivation of either OmpK35 or Omp36 individually reduces the potency of vaborbactam. Inactivation of OmpK35 was associated with an effect much smaller than that associated with the inactivation of OmpK36, indicating that OmpK36 is the preferred porin for vaborbactam uptake. Surprisingly, the effect of the minor porin mutation was still observable in the context of the functional major porin. We hypothesize that there is some interplay between meropenem and vaborbactam uptake. When both porins are open, meropenem and vaborbactam use their preferred channels, while the closure of one porin may result in competition for passage through the single porin. An important finding was a marked reduction of the potency of vaborbactam against strains that lacked both major porins. Another finding was that a variant of OmpK36 that was predicted to have a constricted inner channel as a result of a 2-amino-acid duplication in the L3 loop and that was partially defective in meropenem uptake appeared to be completely defective in the uptake of vaborbactam. Other mutations that affect *ompK36* have been reported in clinical isolates, such as the downregulation of *ompK36* expression due to the insertion of IS*5* in its promoter region (12); the effect of such mutations was not evaluated in this study. Unlike meropenem, vaborbactam does not appear to be a substrate of the AcrAB-TolC efflux pump, since overexpression of AcrAB had a minimal effect on vaborbactam potency, even in the absence of major porins.

This study demonstrated that established drug resistance mechanisms contribute to the potencies of meropenem and vaborbactam alone and in combination. The biggest reduction of meropenem-vaborbactam potency was observed in KPC-producing strains lacking both porins and overexpressing AcrAB. Investigation of the concentration-response for the restoration of meropenem activity by vaborbactam against various KPC-producing strains with these additional mutations indicates that with vaborbactam concentrations of ≥8 μg/ml, the meropenem MICs are ≤2 μg/ml, even for the most resistant strains containing multiple mutations. This result informed studies that were designed to identify the target *in vivo* exposures for both meropenem and vaborbactam required to maximize the efficacy of the combination, overcome the negative impact of preexisting mutations in the envelope genes of Gram-negative bacteria, and minimize the emergence of resistance *de novo*. Recently completed clinical trials demonstrate that these target exposures appear to be achievable due to the excellent safety profiles of both meropenem and vaborbactam (47, 48).

## MATERIALS AND METHODS

### Panels of engineered bacterial strains containing cloned beta-lactamases and various combinations of porin and efflux mutations.

The panel of engineered isogenic strains of E. coli producing individual beta-lactamases was constructed to study the profile of beta-lactamase inhibition by vaborbactam. DNA samples isolated from clinical strains producing various beta-lactamases were used as the templates for PCR with high-fidelity DNA polymerase Vent (New England BioLabs). The PCR primers contained sequences for restriction enzymes to facilitate cloning into vector plasmid pUCP24 (49) at the multiple-cloning site. After digestion with the appropriate enzymes and ligation, the ligation mix was transformed into E. coli DH5α competent cells and transformants were selected on LB agar plates containing gentamicin at 15 μg/ml. After confirmation of the correct clones by restriction digestion and sequencing, selected recombinant plasmids were introduced into E. coli ECM5497 (MG1655) and two K. pneumoniae strains, KPM1001 (ATCC 43816) and KPM1176, by transformation and selection on plates containing gentamicin at the appropriate concentrations. The sequences of the primers used for the amplification of the beta-lactamase genes are provided in Table S1 in the supplemental material. On the basis of sequence analysis (GenBank accession no. CP009208 [50]) KPM1001 is a wild-type strain containing functional genes encoding efflux pumps, such as AcrAB-TolC and the major porins OmpK35 and OmpK36. This strain does not have mutations in known regulators of efflux and porin genes; hence, expression of these genes was considered to be at the wild-type level. KPM1176 is an isogenic derivative of KPM1001 (Table S2) with *acrAB* overexpressed, *ompK35* downregulated (Table S5) due to a mutation in the *ramR* gene (a frameshift at amino acid 46 of RamR), and a loss-of-function mutation in *ompK36* (a frameshift at amino acid 266 of OmpK36).

The panel of engineered isogenic strains of K. pneumoniae with various combinations of porin and efflux mutations with or without KPC was used to evaluate the impact of various molecular determinants on meropenem and meropenem-vaborbactam MICs. Most of the strains are derivatives of wild-type strain KPM1026a, a streptomycin-resistant mutant of KPM1001 (ATCC 43816). All KPC-3-producing strains were constructed by conjugating the plasmid pKpQIL (51) from clinical isolate K. pneumoniae KP1074 (ATCC BAA-2814) into various isogenic derivatives of KPM1026a.

Two clinical KPC-3-producing clinical isolates, KP1074 and KP1004, were used in this study. KP1074 has a nonfunctional OmpK35 due to a frameshift mutation at amino acid 42. It also contains a defective variant of OmpK36 that has a duplication of 2 amino acids, Gly134 and Asp135 (the GD repeat). This duplication in the L3 internal loop of OmpK36 is commonly seen in sequence type 258 (ST258) isolates carrying *bla*_KPC_ and is predicted to functionally constrict the inner channel of the porin (29), causing reduced susceptibility to carbapenems. KPM2644 is a derivative of KP1074 with a complete deletion of the *ompK36* gene. Another clinical isolate, KP1004, similar to KP1074, carries the KPC-producing plasmid pKpQIL. It also has the same frameshift at amino acid 42 in OmpK35 found in KP1074. KP1004 and KP1074 have nearly identical OmpK36 amino acid sequences; the only difference is the absence in KP1004 of the GD repeat in OmpK36 that is found in KP1074. Therefore, OmpK36 in KP1004 is completely functional. A detailed description of all these strains is provided in Table S2.

### Construction of *ompK35* and *ompK36* knockout strains of K. pneumoniae.

Construction of *ompK35* and *ompK36* knockout mutants was carried out following the method described by Datsenko and Wanner (52) with major modifications. As a selectable marker to construct knockout strains, we used resistance to rifampin and the corresponding gene *arr2* since most of the K. pneumoniae strains are resistant to ampicillin or chloramphenicol, the two markers that are usually used to construct knockout strains in various organisms. The clinical isolate KPM1346, which carries the *arr2* gene, was used as the template for PCR. To facilitate future removal of the *arr2* gene from knockout constructs, the FRT sequence was added to each end of the *arr2* gene using primers FRT-arr2-F (5′-ACAGGATCCGAAGTTCCTATTCTCTAGAAAGTATAGGAACTTCCAAGCAGCAAGCGCGTTAC-3′) and FRT-arr2-R (5′-CACGTCGACGAAGTTCCTATACTTTCTAGAGAATAGGAACTTCCTAGTCTTCAATGACGTGTA-3′). BamHI and SalI restriction sites were added to facilitate cloning. The PCR product was digested with BamHI and SalI and cloned into pUC19 that had been digested with the same restriction enzymes. The correct recombinant plasmid transformed into E. coli DH5α was confirmed by DNA sequencing at Eton Biosciences (San Diego, CA). Plasmid pKD46-GM, containing bacteriophage lambda Red recombinase, was transformed into various strains to facilitate *ompK35* and *ompK36* knockout experiments.

To knock out the *ompK35* gene, PCR products carrying the FRT-*arr2* cassette flanked by 54 bp and 57 bp of sequences of the 5′ and the 3′ ends of the *ompK35* coding region, respectively, were prepared by PCR using primers KP-*ompK35*-del-F (5′-ATGAGGGTAATAAATAATGATGAAGCGCAATATTCTGGCAGTGGTGATCCCTGCGTGAATTCGAGCTCGGTACCC-3′) and KP-*ompK35*-del-R (5′-TTAGAACTGGTAAACGATACCCACGGCCGCCTGGTCGTCAGTGGCGACACCAGCCGCTGACCATGATTACGCCAAGC-3′) with pUC19::FRT-*arr2* as the template.

To facilitate the knockout of the *ompK36* gene, the *ompK36* gene coding region and 88 bp of the upstream sequence of strain KPM1026a were first cloned into pUCP24. Then, plasmid pUC19::FRT-*arr2* was digested with SmaI and PstI to obtain the FRT-*arr2* cassette, which was subsequently subcloned into plasmid pUCP24::*ompK36* that had been digested with ScaI and PstI. The resulting plasmid, pUCP24::*ompK36*-FRT-*arr2*, had 874 bp of the *ompK36* coding sequence (positions +15 to +888) deleted and replaced by the FRT-*arr2* cassette. This plasmid was used as a template to generate the PCR product for *ompK36* gene knockout using primers KP-*ompK36*-F (5′-CAGCACAATGAAATAGCCGAC-3′) and KP-ompK36-R (5′-TTAGAACTGGTAAACCAGGC-3′). This PCR product had 102 bp of the *ompK36* gene 5′ region (positions −88 to +14) and the last 207 bp of the coding region (positions +904 to +1110).

The PCR products for the *ompK35* and *ompK36* knockouts were transformed into strains carrying plasmid pKD46-GM by electroporation using a Bio-Rad Gene Pulser apparatus (Hercules, CA). Electrocompetent cells were prepared following the manufacturer's instructions with the following modifications. After electroporation, transformants were selected on LB agar plates containing rifampin (20 mg/liter), and their sequences were confirmed by PCR using primers specific for sequences within and outside the deleted regions of the *ompK35* and *ompK36* genes and the primers specific for the FRT-*arr2* cassette.

### Conjugation experiments.

Strain KP1074 containing plasmid pKpQIL, which carries *bla*_KPC-3_, was used as a donor in conjugation experiments. pKpQIL was introduced into various derivatives of streptomycin-resistant strain KPM1026a with various combinations of efflux and porin mutations. Both donor and recipient strains were grown in LB broth at 37°C with shaking overnight. Fifty microliters of the donor culture was mixed with 50 μl of the recipient culture, followed by centrifugation for 1 min at 5,000 rpm at room temperature. After the supernatant was removed, the cells were resuspended in 50 μl of LB and spotted onto an LB agar plate without antibiotics. The plate was incubated at 37°C for 4 to 5 h to form a bacterial lawn of growth. The cells were harvested and resuspended in 1 ml of LB medium to achieve an optical density at 600 nm (OD_600_) of 0.2 to 0.5. Resuspended cells (0.1 ml) were plated on LB agar plates containing 1,000 μg/ml streptomycin and 8 μg/ml aztreonam to inhibit both parental strains but allow the growth of the transconjugants, which were verified by PCR to contain *bla*_KPC-3_.

### Antimicrobial susceptibility testing.

Bacterial isolates were subjected to broth microdilution susceptibility testing, performed according to Clinical and Laboratory Standards Institute (CLSI) methods (53), using panels prepared in-house. A checkerboard assay conforming to the procedures described by Moody in the *Clinical Microbiology Procedures Handbook* (54) was used to evaluate the effects of various concentrations of vaborbactam on the MICs of various antibiotics. MPC_16_ and MPC_max_ (where MPC stands for the minimal potentiating concentration) values were used to define the potency of vaborbactam. MPC_16_ was defined as the concentration of vaborbactam that was required to reduce the antibiotic MIC 16-fold. MPC_max_ was defined as the concentration of vaborbactam that achieved the maximal effect in increasing antibiotic potency.

Meropenem was purchased from Sandoz; all other antibiotics were from Sigma-Aldrich. Vaborbactam was synthesized at The Medicines Company, San Diego, CA (lot P-232-159-2).

### Determination of expression of efflux and porin genes.

Single colonies from an overnight plate were inoculated into cation-adjusted Mueller-Hinton broth and grown at 37°C until an OD_600_ of 0.7 was obtained. Cell pellets were collected by centrifugation, and total RNA was isolated using an Ambion RiboPure-Bacteria RNA isolation kit (Thermo Fisher, San Diego, CA). The residual DNA in the RNA samples was removed by treatment with DNase I, according to the manufacturer's instructions. Reverse transcription (RT) was performed using a TaqMan reverse transcriptase reagents kit (Thermo Fisher, San Diego, CA). A mixture of reverse primers specific for the genes to be tested, *acrB*, *ompK35*, and *ompK36*, and the internal control housekeeping gene, *rpoB* (Table S1), each at a final concentration of 0.5 μM, was used as RT primers.

The RT reaction mixture was diluted 10-fold and used in a quantitative real-time PCR (qPCR) performed on an ABI Prism 7000 sequencing system (Applied Biosystems) using SYBR Select master mix (Thermo Fisher). For these reactions, 9 μl of the diluted RT reaction mixture was used as the template and mixed with 10 μl of SYBR Select master mix (2×) and 1 μl of a qPCR primer pair mixture (Table S1) to make the final concentration of the forward and reverse primers of 0.5 μM. The qPCR was run with the following thermal cycling conditions: 55°C for 2 min, 95°C for 5 min, and 40 cycles of 95°C for 15 s and 60°C for 1 min. The qPCR results (threshold cycle [*C_T_*] values) were normalized with the housekeeping gene *rpoB* by subtracting the *C_T_* value of the test gene from the *C_T_* value of the *rpoB* gene from the same RT reaction. To calculate the level of expression relative to that by strain KPM1026a, the normalized *C_T_* value of a gene in a test strain was subtracted from that of the same gene in KPM1026a, and the difference (Δ*C_T_*) was used as a logarithmic power (base 2). At least three independent RNA samples isolated from three separate cultures were used to determine the average transcript level of each strain.

## Supplementary Material

Supplemental material
