# Supplementary material for: Vaborbactam: Spectrum of Beta-Lactamase Inhibition and Impact of Resistance Mechanisms on Activity in Enterobacteriaceae
Source: Antimicrob Agents Chemother. 2017 Oct 24;61(11):e01443-17. doi: 10.1128/AAC.01443-17 (PMC5655098; doi:10.1128/AAC.01443-17)
Supplement: Supplemental material [file supp_61_11_e01443-17__index.html]

Supplemental material 

# Vaborbactam: Spectrum of Beta-Lactamase Inhibition and Impact of Resistance Mechanisms on Activity in Enterobacteriaceae

## Supplemental material

- Supplemental file 1 -

  Supplemental Tables S1 to S5

  PDF, 495K
