# Supplementary material for: Vaborbactam: Spectrum of Beta-Lactamase Inhibition and Impact of Resistance Mechanisms on Activity in Enterobacteriaceae
Source: Antimicrob Agents Chemother. 2017 Oct 24;61(11):e01443-17. doi: 10.1128/AAC.01443-17 (PMC5655098; doi:10.1128/AAC.01443-17)
Supplement: Supplemental material [file AAC.01443-17_zac011176634s1.pdf]

Control number: AAC01443-17

Manuscript title: Vaborbactam: Spectrum of Beta-Lactamase Inhibition and Impact of Resistance Mechanisms on Activity in Enterobacteriaceae

Table S1. Primer sequences used in cloning various beta-lactamase genes and in reverse transcription-quantitative PCR (RT-qPCR)

**Cloning of beta-lactamase genes**				
**Source strain**	**Cloned beta-lactamase**	**Primer name**	**Primer sequence (5' to 3')**	**Restriction site added**
KP1004	KPC-2	KPC-2-own-F	ACACGAATTC CAAGGAATATCGTTGATGTCACTGTATCGCCGTC	*Eco*RI
		KPC-2-R1	ACACAAGCTT TACTGCCCGTTGACGCCCAA	*Hind*III
EC1007	KPC-3	KPC-2-own-F	ACACGAATTC CAAGGAATATCGTTGATGTCACTGTATCGCCGTC	*Eco*RI
		KPC-2-R1	ACACAAGCTT TACTGCCCGTTGACGCCCAA	*Hind*III
SM1000	SME-2	SME-2-F1	ACACGGATCC AGGAGGTTAATTCTGATGTCAAACAAAG	*Bam*HI
		SME-2-R1	ACACAAGCTT TTAATCAATTGCCTGAATTG	*Hind*III
ECL1004	NMC-A	NMC-A-F2	ACGCGAATTC ATATAAGGTAAAACCATGTCACTTAATGTAAAGC	*Eco*RI
		NMC-A-R1	ACACGGATCC TTATTTAAGGTTATCAATTGC	*Bam*HI
ECM6625	SHV-5	SHV-12-F2	ACGCGAATTC GGATGTATTGTGGTTATGCGTTATATTCGCCTGTG	*Eco*RI

		SHV-12-R1	ACACAAGCTT TTAGCGTTGCCAGTGCTCGA	*Hind*III
KP1010	SHV-12	SHV-12-F2	ACGCGAATTC GGATGTATTGTGGTTATGCGTTATATTCGCCTGTG	*Eco*RI
		SHV-12-R1	ACACAAGCTT TTAGCGTTGCCAGTGCTCGA	*Hind*III
KP1012	SHV-18	SHV-12-F2	ACGCGAATTC GGATGTATTGTGGTTATGCGTTATATTCGCCTGTG	*Eco*RI
		SHV-12-R1	ACACAAGCTT TTAGCGTTGCCAGTGCTCGA	*Hind*III
ECM6619	TEM-10	TEM-26-F1	ACGCGAATTC GAAAAAGGAAGAGTATGAGTATTCAACATTTCCG	*Eco*RI
		TEM-26-R1	ACACAAGCTT TACCAATGCTTAATCAGTGAGGC	*Hind*III
ECM6621	TEM-26	TEM-26-F1	ACGCGAATTC GAAAAAGGAAGAGTATGAGTATTCAACATTTCCG	*Eco*RI
		TEM-26-R1	ACACAAGCTT TACCAATGCTTAATCAGTGAGGC	*Hind*III
EC1008	CTX-M-3	CTX-M-3-F2	ACGCGAATTC AGAATAAGGAATCCCATGGTTAAAAAATCACTGCG	*Eco*RI
		CTX-M-3-R1	ACACAAGCTT TTACAAACCGTCGGTGACG	*Hind*III
KP1005	CTX-M-14	CTX-M-14-F1	ACGCGAATTC AGGAGGTTAATTCTGATGGTGACAAAGAGAGTGCA	*Eco*RI
		CTX-M-14-R1	ACACAAGCTT TTACAGCCCTTCGGCGATG	*Hind*III
KP1009	CTX-M-15	CTX-M-15-F1	ACACGAATTC AGGAGGTTATCGTTGATGGTTAAAAAATCACTGCG	*Eco*RI
		CTX-M-15-R1	ACACAAGCTT TTACAAACCGTTGGTGACGA	*Hind*III
EC1014	DHA-1	DHA-1-F1	ACGCGAATTC GGAAGGTTAATTCTGATGAAAAAATCGTTATCTGC	*Eco*RI
		DHA-1-R1	ACACAAGCTT TTATTCCAGTGCACTCAAAATAG	*Hind*III
EC1016	FOX-5	FOX-5-F2	ACGCGAATTC CACGAGAATAGCCATATGCAACAACGGCGTGCG	*Eco*RI
		FOX-5-R1	ACACAAGCTT TCACTCGGCCAACTGACTCA	*Hind*III

ECL1002	P99	P99-F2	ACGCGAATTC GACTCGCTATTACGGAAGAT	*Eco*RI
		P99-HIS-R2	ACACAAGCTT TTAGTGGTGATGATGGTGATGCTGTAGCGCCTCGAGGA	*Hind*III
KP1013	CMY-2	CMY-2-F2	ACGCGAATTC TACGGAACTGATTTCATGATGAAAAAATCGTTATGC	*Eco*RI
		CMY-2-R1	ACACAAGCTT TTATTGCAGCTTTTCAAGAATGC	*Hind*III
KX1000	OXA-2	OXA-2-F2	ACACGGATCC ATTAAGGAAAAGTTAATGGCAATCCGAATCTTCGC	*Bam*HI
		OXA-2-R1	ACACAAGCTT TTATCGCGCAGCGTCCGAGT	*Hind*III
KP1007	OXA-10	Oxa-10-F2	ACACGAATTC CACCAAGAAGGTGCCATGAAAACATTTGCCGCAT	*Eco*RI
		Oxa-10-R1	ACACAAGCTT TTAGCCACCAATGATGCCCT	*Hind*III
EC1062	OXA-48	Oxa-48-F2	ACACGAATTC AAGCAAGGGGACGTTATGCGTGTATTAGCC	*Eco*RI
		Oxa-48-HIS-R1	ACACAAGCTT CTAGTGGTGATGATGGTGATGGGGAATAATTTTTTCCTG	*Hind*III
KP1081	NDM-1	NDM1-F2	ACACGAATTC GCTGAATAAAAGGAAAACTTG	*Eco*RI
		NDM1-HIS-R1	ACACAAGCTT TCAGTGGTGATGATGGTGATGGCGCAGCTTGTCGGCCA	*Hind*III
KP1014	VIM-1	VIM-1-F2	ACGCGAATTC CCCTATGGAGTCTTGATGTTAAAAGTTATTAGTAG	*Eco*RI
		VIM-1-R1	ACACAAGCTT CTACTCGGCGACTGAGCGATT	*Hind*III
Pa1066	VIM-2	VIM-2-own-F	ACACGAATTC ACAAAGTTATGCCGCACTC	*Eco*RI
		VIM-2-P24-R	ACATAAGCTT CTACTCAACGACTGAGCGAT	*Hind*III
Pa1070	VIM-7	VIM-7-own-F	ACACGGATCC ACAAAGTTATCGCAGTCGG	*Bam*HI
		VIM-7-P24-R	ACATAAGCTT ACTCGGCCACCGGGCGTACTTT	*Hind*III

Pa1068	SPM-1	SPM-1-own-F1	ACACGAATTC TTATCGGAGATCGGAATGAAC	*Eco*RI
		SPM-1-P24-R1	ACACAAGCTT CTACAGTCTCATTTCGCCAA	*Hind*III
Pa1069	GIM-1	GIM-1-own-F	ACACGGATCC AGTTAGAAGGATGATTTCC	*Bam*HI
		GIM-1-P24-R	ACATCTGCAG TTAATCAGCCGACGCTTCAGC	*Pst*I
Pa1064	IMP-13	PA-IMP-own-F	ACGCGAATTC TAGAAAAGGDWARGTATGAA	*Eco*RI
		PA-IMP-R	ACGCGGATCC GTTAGAAAWTTAGYTACTTGG	*Bam*HI
Pa1067	IMP-15	PA-IMP-own-F	ACGCGAATTC TAGAAAAGGDWARGTATGAA	*Eco*RI
		PA-IMP-R	ACGCGGATCC GTTAGAAAWTTAGYTACTTGG	*Bam*HI
Pa1065	IMP-18	PA-IMP-own-F	ACGCGAATTC TAGAAAAGGDWARGTATGAA	*Eco*RI
		PA-IMP-R	ACGCGGATCC GTTAGAAAWTTAGYTACTTGG	*Bam*HI
ECM6846	CcrA	BF-ccrA-own-F	ACGCGAATTC ATATAAAAGAATAAAATGAAAACAGTATTTATCC	*Eco*RI
		BF-ccrA-R	ACGCAAGCTT CTATGGCTTTGAAGTGCTTTC	*Hind*III
SMM1020	L1	SM-K279a-L1-own-F	ACACGAATTC AAGCGGACGTGGATCATGCGTTTTACCCTGCTCGC	*Eco*RI
		SM-K279a-L1-R	ACATAAGCTT TCAGCGGGTCCCGGCCGTTT	*Hind*III

Reverse transcription-quantitative PCR

Primer	Gene	Primer sequence (5' to 3')	Used for
KP-acrB-qF2	*acrB*	CCGGTATCGCGTTCGTTTCGC	qPCR
KP-acrB-qR2		AGTTTTTCGTGGCCCAGACCCG	RT; qPCR

KP-ompK35-qF	*ompK35*	CAACCAGCTGGACGACAACGA	qPCR
KP-ompK35-qR		AGAATTGGTAAACGATACCCACG	RT; qPCR
KP-ompK36-qF	*ompK36*	GTGGTTGCTCAGTACCAGTTC	qPCR
KP-ompK36-qR		GCTGTTGTCGTCCAGCAGGTTG	RT; qPCR
KP-rpoB-qF	*rpoB*	AAGGCGAATCCAGCTTGTTCAGC	qPCR
KP-rpoB-qR		TGACGTTGCATGTTCGCACCCATCA	RT; qPCR

All source strains are part of the Medicines Company strain collection. Abbreviations of species that served as a source of cloned genes: KP, *Klebsiella pneumoniae*; SM, *Serratia marcescens*; ECL, *Enterobacter cloacae*, ECM or EC, *Escherichia coli*; KX, *Klebsiella oxytoca*; Pa, *Pseudomonas aeruginosa*; SMM, *Stenotrophomonas maltophilia*.

Sequences corresponding to restriction sites are underlined.

qPCR, quantitative PCR; RT, reverse transcription

Table S2. The panel of isogenic strains with various combinations of efflux and porin mutations and clinical strains used in this study

Strains	Beta-lactamase	Parent strain/Recipient	Relevant genotype	Construction/ selection/ source
KPM1001 (ATCC 43816)	SHV-24[1]	NA	Wild type	ATCC
KPM1026a	SHV-24	KPM1001	Wild type	Selected from ATCC 43816 on 200 µg/ml of streptomycin, K88R in S12
KPM1271	SHV-24, KPC-3, TEM-1[2]	KPM1026a	Wild type	Conjugation of pKpQIL with bla_{KPC-3} from KP1074 into KPM1026a
KPM1004	SHV-24	ATCC 43816	*ramR*	Selected from ATCC 43816 on 4 µg/ml of tigecycline,8bp insertion in *ramR* causing a frame-shift from amino acid no.46
KPM1027	SHV-24	KPM1004	*ramR*	Selected from KP1027 on 200 µg/ml of streptomycin, K43T in S12
KPM1272	SHV-24, KPC-3, TEM-1	KPM1027	*ramR*	Conjugation of pKpQIL with bla_{KPC-3} from KP1074 into KPM1027
KPM1176	SHV-24	KPM1027	*ramR, ompK36_1176*	Selected from KPM1027 on 0.25 µg/ml of meropenem; carries frame-shift from amino acid no. 266 in OmpK36
KPM2067	SHV-24, KPC-3, TEM-1	KPM1271	*ompK36_2067*	Selected from KPM1271 on meropenem 2 µg/ml and vaborbactam 2 µg/ml; frame-shift from amino acid no. 54 of OmpK36
KPM2040	SHV-24	KPM2067	*ompK36_2067*	pKpQIL plasmid with bla_{KPC-3} cured from KPM2067
KPM2600	SHV-24	KPM1026a	*ΔompK35*	*ompK35* disrupted in KPM1026a
KPM2601	SHV-24, KPC-3, TEM-1	KPM2600	*ΔompK35*	Conjugation of pKpQIL with blaKPC-3 from KP1074 into KPM2600
KPM2610	SHV-24	KPM1027	*ramR ΔompK35*	*ompK35* disrupted in KPM1027
KPM2828	SHV-24, KPC-3, TEM-1	KPM2610	*ramR ΔompK35*	Conjugation of pKpQIL with bla_{KPC-3} from KP1074 into KPM2610
KPM2613	SHV-24	KPM2040	*ompK36_2040 ΔompK35*	*ompK35* disrupted in KPM2040
KPM2631	SHV-24, KPC-3, TEM-1	KPM2613	*ompK36_2040 ΔompK35*	Conjugation of pKpQIL with bla_{KPC-3} from KP1074 into KPM2613

KPM2966	SHV-24	KPM2613	*ramR ompK36_2040 ΔompK35*	Selected from KPM2613 on 2 μg/ml of tigecycline, TAA at amino acid no. 164 of RamR
KPM2965	SHV-24, KPC-3, TEM-1	KPM2631	*ramR ompK36_2040 ΔompK35*	Selected from KPM2631 on 4 μg/ml of tigecycline,8bp insertion in *ramR* causing a frame-shift from amino acid no. 50
KPM2592	SHV-24	KPM1026a	*ΔompK36*	*ompK36* disrupted in KPM1026a
KPM2599	SHV-24, KPC-3, TEM-1	KPM2592	*ΔompK36*	Conjugation of pKpQIL with bla_{KPC-3} from KP1074 into KPM2592
KPM2658	SHV-24	KPM1027	*ramR ΔompK36*	*ompK36* disrupted in KPM1027
KPM2818	SHV-24, KPC-3, TEM-1	KPM2658	*ramR ΔompK36*	Conjugation of pKpQIL with bla_{KPC-3} from KP1074 into KPM2658
KP1074	SHV-11[3], KPC-3, TEM-1	Clinical isolate	*ompK35*_fs42[4]*ompK36*_GD[5]	The Medicines Company strain collection
KPM1308	SHV-11	KPM1074	*ompK35*_fs42*ompK36*_GD	pKpQIL plasmid cured from KP1074 and streptomycin resistant mutant was selected
KPM2617	SHV-11	KPM1308	*ompK35*_fs42 *ΔompK36*	*ompK36* disrupted in KPM1308
KPM2644	SHV-11, KPC-3, TEM-1	KPM2617	*ompK35*_fs42 *ΔompK36*	Conjugation of pKpQIL with bla_{KPC-3} from KP1074 into KPM2617
KP1004	SHV-11, KPC-3, TEM-1	Clinical isolate	*ompK35*_fs42 *ompK36* wild-type	The Medicines Company strain collection

[1]SHV-24 is a chromosomally encoded beta-lactamase in all ATCC 43816/KPM1026a derivatives

[2]The *bla*TEM-1 gene is located on pKpQIL plasmid along with bla_{KPC-3}

[3]SHV-11 is a chromosomally encoded beta-lactamase in KP1074 and KP1004.

[4] fs42, frame-shift in OmpK35 at amino acid no. 42 that results in non-functional protein

[5] Duplication of two amino acids, Gly134 and Asp135, located within the L3 internal loop and associated with the reduced susceptibility to carbapenems due to constriction of the channel (29).

Table S3. The effect of varying vaborbactam concentrations on aztreonam MICs in *E. coli* strains expressing various cloned beta-lactamases

		**Aztreonam MIC (μg/ml) in the presence of varied concentrations of vaborbactam (μg/ml)**								
**Strain**	**Beta-Lactamase**	***0***	***0.15***	***0.3***	***0.6***	***1.25***	***2.5***	***5***	***10***	**MPC_{16}**
ECM6701	KPC-2	16	0.25	≤0.125	≤0.125	≤0.125	≤0.125	≤0.125	≤0.125	**<0.15**
ECM6702	KPC-3	32	0.5	0.25	≤0.125	≤0.125	≤0.125	≤0.125	≤0.125	**<0.15**
ECM6696	NMC-A	32	2	1	0.5	0.25	≤0.125	≤0.125	≤0.125	**≤0.15**
ECM6706	SME-2	>128	4	2	1	0.5	0.25	0.25	≤0.125	**<0.15**
ECM6695	CTX-M-3	4	1	0.5	0.25	≤0.125	≤0.125	≤0.125	≤0.125	**0.6**
ECM6693	CTX-M-14	4	1	0.5	0.25	0.25	≤0.125	≤0.125	≤0.125	**0.6**
ECM6694	CTX-M-15	8	8	4	2	1	0.5	0.25	≤0.125	**2.5**
ECM6718	SHV-5	16	8	4	2	2	1	0.5	0.25	**2.5**
ECM6698	SHV-12	32	32	16	16	8	8	4	2	**10**
ECM6699	SHV-18	8	8	8	4	2	1	0.5	0.25	**5**
ECM6713	TEM-10	32	32	32	32	16	8	8	2	**10**
ECM6714	TEM-26	8	8	4	4	2	2	1	1	**>10**
ECM6700	CMY-2	4	2	2	2	1	0.5	0.25	≤0.125	**5**
ECM6715	AmpC-ECL (P99-like)	8	8	8	4	2	1	0.5	0.25	**5**
ECM6691	MIR-1	16	8	4	2	1	0.5	0.5	0.25	**1.25**

MPC, minimal potentiation concentration, MPC_{16}, concentration of vaborbactam required to reduce MIC 16-fold.

Vaborbactam concentrations are in bold italic.

The effect of increasing vaborbactam concentrations on aztreonam MICs in a panel of *E. coli* clones was determined. The concentration of vaborbactam required to reduce the MIC 16-fold (minimal potentiation concentration, MPC_{16}) was used as a measure of vaborbactam potency. Table S3 shows that vaborbactam had the highest inhibitory potency in strains producing KPC compared to other class A or C beta-lactamases; the MPC_{16} for these class A carbapenemase-producing strains was ≤0.15 µg/ml. Vaborbactam was 8- to 32-fold less potent in strains producing ESBLs; the MPC_{16} ranged from 0.6 to 2.5 µg/ml, from 1.25 to 5 µg/ml and from 5 to >10 µg/ml for CTX-M-, class C- and SHV/TEM-producing strains, respectively.

Table S4. MICs of ceftazidime alone or in combination with vaborbactam against the panel of engineered *E. coli* strains producing various cloned metallo-beta-lactamases

		Ceftazidime MIC (µg/ml)	
**Strain**	**Beta-lactamase**	**alone**	**w/vaborbactam at 8 µg/ml**
ECM6704	none	**≤0.125**	**≤0.125**
ECM6909	VIM-2	**2**	2
ECM6903	VIM-7	**0.25**	0.25
ECM6865	SPM-1	**1**	1
ECM6912	GIM-1	**16**	16
ECM6784	IMP-13	**64**	64
ECM6785	IMP-18	**>64**	>64
ECM6786	IMP-15	**64**	64
ECM6852	CcrA1	**32**	32
ECM6853	L1	**32**	32

Table S5. Relative expression of *acrB*, *ompK35* and *ompK36* in KPM1026a and its derivatives, KPM1027 and KPM1176

	**Strains**		
**Genes**	**KPM1026a**	**KPM1027[2] (ave±stdev)**	**KPM1176**
ompK35	1.00	0.14±0.02	0.14
ompK36	1.00	0.94±0.43	0.44
acrB	1.00	3.11±0.64	3.88
rpoB[1]	1.00	1.00	1.00

[1] The expression of *acrB*, *ompK35* and *ompK36* in each strain was normalized with the housekeeping gene *rpoB*. To calculate the level of expression relative to strain KPM1026a, the normalized CT value of a gene in a test strain was subtracted from that of the same gene in KPM1026a, and the difference (ΔCT) was used as a logarithmic power (base=2).

[2] Data for KPM1027 is the average of 3 tests.
